# Phosphatase of regenerating liver 3 (PRL-3) is overexpressed in human prostate cancer tissue and promotes growth and migration

**DOI:** 10.1186/s12967-016-0830-z

**Published:** 2016-03-15

**Authors:** Esten N. Vandsemb, Helena Bertilsson, Pegah Abdollahi, Øystein Størkersen, Thea Kristin Våtsveen, Morten Beck Rye, Torstein Baade Rø, Magne Børset, Tobias S. Slørdahl

**Affiliations:** K. G. Jebsen Center for Myeloma Research, Norwegian University of Science and Technology, PO Box 8905, 7491 Trondheim, Norway; Department of Cancer Research and Molecular Medicine, Norwegian University of Science and Technology, Trondheim, Norway; Department of Urology, St Olavs University Hospital, Trondheim, Norway; Department of Pathology, Trondheim University Hospital, Trondheim, Norway; Department of Pediatrics, St Olavs University Hospital, Trondheim, Norway; Department of Immunology and Transfusion Medicine, St Olavs University Hospital, Trondheim, Norway; Clinic of Medicine, St Olavs University Hospital, Trondheim, Norway

**Keywords:** PTP4A3, Oncogenesis, Prostate cancer, Molecular pathogenesis, PRL-3

## Abstract

**Background:**

PRL-3 is a phosphatase implicated in oncogenesis in multiple cancers. In some cancers, notably carcinomas, PRL-3 is also associated with inferior prognosis and increased metastatic potential. In this study we investigated the expression of *PRL*-*3* mRNA in fresh-frozen samples from patients undergoing radical prostatectomy because of prostate cancer (PC) and the biological function of PRL-3 in prostate cancer cells.

**Methods:**

Samples from 41 radical prostatectomy specimens (168 samples in total) divided into low (Gleason score ≤ 6), intermediate (Gleason score = 7) and high (Gleason score ≥ 8) risk were analyzed with gene expression profiling and compared to normal prostate tissue. *PRL*-*3* was identified as a gene with differential expression between healthy and cancerous tissue in these analyses. We used the prostate cancer cell lines PC3 and DU145 and a small molecular inhibitor of PRL-3 to investigate whether PRL-3 had a functional role in cancer. Relative ATP-measurement and thymidine incorporation were used to assess the effect of PRL-3 on growth of the cancer cells. We performed an in vitro scratch assay to investigate the involvement of PRL-3 in migration. Immunohistochemistry was used to identify PRL-3 protein in prostate cancer primary tumor and corresponding lymph node metastases.

**Results:**

Compared to normal prostate tissue, the prostate cancer tissue expressed a significantly higher level of *PRL*-*3*. We found PRL-3 to be present in both PC3 and DU145, and that inhibition of PRL-3 led to growth arrest and apoptosis in these two cell lines. Inhibition of PRL-3 led to reduced migration of the PC3 cells. Immunohistochemistry showed PRL-3 expression in both primary tumor and corresponding lymph node metastases.

**Conclusions:**

PRL-3 mRNA was expressed to a greater extent in prostate cancer tissue compared to normal prostate tissue. PRL-3 protein was expressed in both prostate cancer primary tumor and corresponding lymph node metastases. The results from our in vitro assays suggest that PRL-3 promotes growth and migration in prostate cancer. In conclusion, these results imply that PRL-3 has a role in the pathogenesis of prostate cancer.

**Electronic supplementary material:**

The online version of this article (doi:10.1186/s12967-016-0830-z) contains supplementary material, which is available to authorized users.

## Background

Phosphatase of regenerating liver (PRL)-3 (gene name *PTP4A3*) is a dual-specificity phosphatase, and thus able to dephosphorylate both tyrosine and serine/threonine residues. Increased interest in the PRL family was triggered in 2001, when Saha et al. [1] conducted a study on colorectal cancer, and found PRL-3 to be overexpressed in liver metastases, but not in the primary tumor nor the normal colon tissue. In recent years, additional studies have investigated PRL-3 in different cancers and found association between the expression of PRL-3 and both metastatic potential and poor prognosis. Similar results have been found in a wide variety of cancers, including colorectal cancer [2–4], breast cancer [5], cervical cancer [6], gastric cancer [7–9], ovarian cancer [10], lung cancer [11] and AML [12].

The morbidity and mortality of cancer is closely related to the spread of metastases. In this process, the tumor cells go through several cellular transformations to be able to colonize distant sites. A key feature of the transformation is the ability to migrate. This ability is acquired as the cell undergoes an epithelial-mesenchymal transition (EMT), which is essential for metastatic dissemination. Several studies have shown a connection between PRL-3 and EMT [13–16], indicating that PRL-3 could have a role in EMT. The process of metastasis is not fully understood, and the definitive role of PRL-3 remains obscure.

Prostate cancer (PC) is the second most common cancer among males worldwide, and the most common cancer among males in developed countries [17]. It is a very heterogeneous disease, spanning from indolent cancer with excellent survival, to a highly aggressive disease with rapid metastatic spread to lymphatic glands and bone. An integrated analysis of copy number alterations and expression, in combination with data from online databases (e.g. Oncomine), identified PRL-3 as a possible factor for tumor progression [18]. The evidence supporting the involvement of PRL-3 in cancer metastasis is growing rapidly, but when it comes to PRL-3 and mechanisms in prostate cancer, the literature is limited. Here we present data elucidating some of the mechanisms PRL-3 could be involved in, and thus contribute to oncogenesis in prostate cancer. A better understanding of the molecular mechanisms of prostate cancer metastasis is of utmost importance for the development of improved therapeutic strategies and better risk stratification of patients with this malignancy. In this study, we hypothesized that PRL-3 is present in prostate cancer and that it has a functional role in disease progression. Hence, we studied whether *PRL*-*3* mRNA and protein were present in fresh-frozen prostate samples from patients operated with radical prostatectomy, and whether it had an effect on proliferation and migration in prostate cancer cell lines.

## Methods

### Cells and reagents

We used the human prostate cancer cell lines PC3 and DU145 (from ATCC). DU145 was grown in Dulbecco’s Modified Eagle Medium (DMEM), and PC3 in Roswell Park Memorial Institute medium-1640 (RPMI), supplemented with 2 mmol/L l-glutamine, 40 µg/mL gentamicin and 10 % heat-inactivated fetal calf serum (FCS). The cell lines were cultured at 37 °C in a humidified atmosphere with 5 % CO_2_. Trypsin was used prior to experiments and culturing for 8–10 min to detach the adherent cells from the plastic flasks and plates. The cells were subcultured twice a week. Cells were washed with Hanks’ balanced salt solution (HBSS) (Sigma-Aldrich, St. Louis, MO, USA). PRL-3 inhibitor I (5-[[5-Bromo-2-[(2-bromophenyl)methoxy]phenyl]methylene]-2-thioxo-4-thiazolidinone) was from Sigma-Aldrich (St. Louis, MO, USA). Dimethyl sulfoxide (DMSO) controls were included since the inhibitor was dissolved in DMSO. The antibodies against PRL-3 (ab50276) and GAPDH were both from Abcam (Cambridge, UK).

### Gene expression profiling

A total of 156 samples were extracted from 41 fresh-frozen slices from patients undergoing radical prostatectomy at the St. Olavs Hospital–Trondheim University Hospital. Patients planned for radical prostatectomy were invited to donate tissue and sign an informed consent form prior to surgery. The Regional Committee for Medical Research Ethics in Central Norway (REC Central) approved the collection of samples. The samples were stained with hematoxylin and eosin and scored according to the Gleason Grading system by a pathologist trained in uropathology, and divided into normal (n = 40), low grade (Gleason score = 6, n = 38), intermediate grade (Gleason score = 7, n = 42) and high grade (Gleason score ≥ 8, n = 36). Cancerous samples were selected from index tumor, and the samples with benign histology were taken as far from index tumor as possible. RNA was extracted manually with mirVana miRNA Isolation Kit (Ambion Inc.). Illumina TotalPrep RNA amplification Kit (Ambion Inc.) was used for amplification of RNA for hybridization. Total RNA from each sample was used to synthesize first-strand cDNA with reverse transcription. After synthesis of second-strand cDNA and purification, cRNA was synthesized via in vitro transcription for 12 h. Illumina Human HT-12 v4 Expression BeadChip (Illumina) was used to measure gene expression. The Minimum Information about a Microarray Gene Experiment (MIAME) guidelines were followed, and the microarray data prepared in a fitting format. Individual cancer and stroma contributions to gene expression was assessed by creating two sample groups where the average stroma content are maximized and minimized between cancer and normal samples (Rye et al., submitted and Additional file 1). The same strategy was also applied to an independent dataset [19] for validation. Our data can be found in array express with accession number: E-MTAB-1041. Method used for fresh tissue harvesting, sample extraction and gene expression analysis is more thoroughly explained by Bertilsson et al. [20, 21].

### Measurement of *PRL*-*3* mRNA with real-time PCR

Non-stimulated cells were washed 4× with HBSS prior to RNA isolation. The RNeasy Mini Kit (Qiagen, Crawley, UK) was used to isolate total RNA from PC3 and DU145 cell lines. Synthesis of cDNA was done by reverse-transcription of 1.0 µg total RNA, using the High Capacity RNA-to-cDNA kit (Life Technologies, Carlsbad, CA, USA), by applying oligo (dT) primers. PTP4A3 (Hs00754750_m1) TaqMan^®^ primer was used to detect gene expression (Life Technologies, Carlsbad, CA, USA). The ΔΔCT-method was used for quantification using GAPDH (Hs99999905_m1) as endogenous reference.

### Immunoblotting

Cells were treated as indicated, collected, pelleted and resuspended in lysis buffer: 1 % NP40, 150 mmol/L NaCl, 50 mmol/L TrisHCl pH 7.5, 10 % glycerol, 1 mmol/L NaF, 2 mmol/L Na_3_VO_4_ and a protease-phosphatase inhibitor mixture (complete mini tablets, Roche, Basel, Switzerland). After 30 min on ice, the nuclei were pelleted by centrifugation at 12,000×*g*, 4 °C for 20 min. Samples were mixed with lithium dodecyl sulfate sample buffer (Invitrogen) with 10 mmol/L dithiothreitol, heated for 10 min at 70 °C and separated on 10 % Bis–tris gels (Invitrogen). Proteins were then transferred to a nitrocellulose membrane with iBlot^®^ dry blotting system (Invitrogen). After blocking the membranes with 5 % BSA in TRIS-buffered saline containing 0.05 % Tween 20 (TBS-T), the membranes were incubated with antibodies against phosphorylated proteins for 1–3 days at 4 °C. Horseradish peroxidase-conjugated antibodies (DAKO Cytomation, Copenhagen, Denmark) and Supersignal^®^ West Femto Maxiumum Sensitivity Substrate (Thermo scientific, Rockford, IL, USA) were used to detect protein signal. The membranes were stripped at 60 °C for 30 min with gentle rotation in stripping buffer containing 62.5 mmol/L Tris–HCl (pH 6.6), 2 % SDS, and 10 mmol/L 2-mercaptoethanol, then washed in Tris-buffered saline with 0.05 % Tween 20, blocked with 5 percent dry milk in TBS-T, and probed with antibodies against GAPDH. Images were taken with LI-COR Odyssey Fc (LI-COR Biosciences, Lincoln, NE, USA).

### Relative ATP-measurement

The relative rate of cell proliferation was estimated by measuring the content of ATP present in the wells using CellTiter-Glo Luminescent (CTG) Cell Viability Assay (Promega, Madison, WI, USA). We followed the instructions provided by the manufacturer. In short, PC3 (20,000) and DU145 (15,000) cells were seeded in quintuples in a 96-well plate and incubated in RPMI/DMEM containing 10 % FCS, with various concentrations of PRL-3 inhibitor I or DMSO for 72 h at 37 °C in a 5 % CO_2_ atmosphere. The provided assay reagent was then added to the plates, after which the plates were agitated on a microplate shaker for 2 min, and kept at room temperature for 10 min before we determined the luminescence. The luminescent signal was recorded with Victor3 plate reader and Wallac 1420 Work Station software (PerkinElmer Inc., Waltham, MA, USA).

### Thymidine incorporation

The prostate cancer cell lines PC3 and DU145 were washed in HBSS four times before seeded into 96-well culture plates with 10 % FCS in RPMI or DMEM, respectively. Next, the cells were treated with various concentrations of the PRL-3 inhibitor I or DMSO. After 56 h, cells were pulsed with 1 µCi of methyl-[3H]-thymidine (NEN Life Science Products, Boston, MA, USA) per well and harvested 16 h later with a Micromate 96-well harvester (Packard, Meriden, CT, USA). The radiation was then measured with a Matrix 96 β counter (Packard).

### Flow cytometry

Viability and apoptosis were investigated by evaluating annexin V-FITC binding and propidium iodide (PI) uptake (APOPTEST-FITC kit; Nexis Research, Kattendikje, The Netherlands) with flow cytometry. The cells were trypsinized before being respuspended, washed 4 times in HBSS, and seeded in 24-well culture plates in 1 mL medium with either RPMI or DMEM (PC3 or DU145, respectively) containing 10 % FCS. Cells were then treated with various concentrations of PRL-3 inhibitor I as indicated. After 72 h, the cells were harvested and washed in PBS. After washing, the cells were resuspended in 300 μL binding buffer, added 0.25 μL annexin V-fluorescin isothiocyanate (FITC), and incubated on ice for 1 h in the dark. Next, PI was added 5 min prior to analysis. BD LSRII Flow Cytometer (BD Biosciences, Franklin Lakes, NJ, USA) was used to classify cells as annexin V-FITC and/or PI-negative or -positive. Cells negative for both PI and annexin V were considered live cells.

### Fluorescence in situ hybridization (FISH)

FISH was done according to standard procedure using metaphases [22]. The *PRL*-*3* probe was prepared from BAC clone RPCI-11953B20 containing the whole *PRL*-*3* gene (Invitrogen) and labeled with SpectrumOrange dUTP using nick translation (Vysis). Whole chromosome paint for chromosome 8 in green (Applied Spectral Imaging, Micro System AB, Spånga, Sweden) was used to determine if the gene was translocated to another chromosome.

### In-vitro scratch assay

10 × 10^5^ PC3 cells were seeded in 1.5 mL media in each well into a 6-well plate. The cells were grown to a confluent layer (48 h), and then a scratch was made in each well by using a pipette tip. Subsequently, the cells were washed gently with PBS, and then various concentrations of PRL-3 inhibitor I in serum-free media were added to the wells. Start picture was taken at time point 0. The cells were then incubated at 37 °C in a 5 % CO_2_ atmosphere, and new pictures were taken after 16 h. The 16 h time point was chosen to decrease the potential impact of proliferation on the closing of the scratch. ImageJ from NIH was used to standardize and present the results.

### Immunohistochemistry (IHC)

Samples (4 µm sections) from paraffin-embedded tissue blocks were pretreated in target retrieval solution (pH = 9), Dako (K8004) in PT Link (Dako, Oslo, Norway) for 20 min at 97 °C. Subsequently, the samples were incubated with antibody from Abcam (ab50276) against PRL-3 (diluted 1:300) for 40 min in room temperature. The detection system used was EnVision/HRP Rabbit, Dako (K4011).

## Results

### Tissue microarray data showed a significant difference between normal and cancerous samples in regards to the expression of *PRL*-*3*

**Fig. 1:**
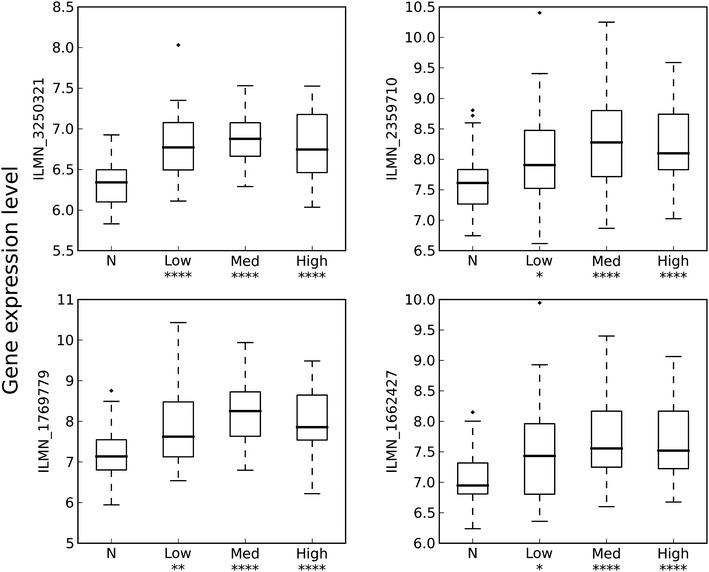
Overexpression of *PRL*-*3* in prostate cancer tissue samples compared to normal tissue from the same samples. A total of 156 samples from radical prostatectomies were divided in groups based on Gleason score (GS), N (normal tissue, 40 samples), low risk (GS ≤ 6, 38 samples), medium risk (GS = 7, 42 samples) and high risk (GS ≥ 8, 36 samples). The groups were analyzed with Illumina gene expression microarray. The panels represent four different *PRL*-*3* probes. For all Gleason groups there was a significant difference in gene expression compared with the normal group. *Asterisks* indicates P ≤ 0.05, *Two* *asterisks* indicates P ≤ 0.01 and *four asterisks* indicates P ≤ 0.0001

A total of 156 samples from 41 different patients were examined, 40 of the samples were classified as having a normal histology. Of the 116 cancer samples, 38 were classified as low risk cancer (Gleason score ≤ 6), 42 samples showed intermediate risk (Gleason score = 7) and 36 samples were classified as high risk cancer (Gleason score ≥ 8). When investigating these samples for expression of *PRL*-*3*, four different probes were used. As shown in Fig. 1, we found a significant difference in *PRL*-3 expression between normal samples and samples containing cancerous tissue. We did not see any significant difference between the groups with different Gleason-score. P values for the different groups compared to normal are given in the supplementary file. In addition, expression differences in PRL-3 improved for all four probes when effects of confounding stroma in the tissue samples were separated from differences related to cancer (see Additional file 1). The same trend was also observed for both probes in an independent dataset. Both datasets ranked PRL-3 among the top 125 cancer related genes after adjusting for stroma effects. Taken together, the results of these tissue microarray studies suggest that PRL-3 plays a significant role in the pathogenesis of prostate cancer.

### PRL-3 was expressed in prostate cancer cell lines PC3 and DU145 and the cell lines harbored multiple copies of the *PRL*-*3* gene locus

**Fig. 2:**
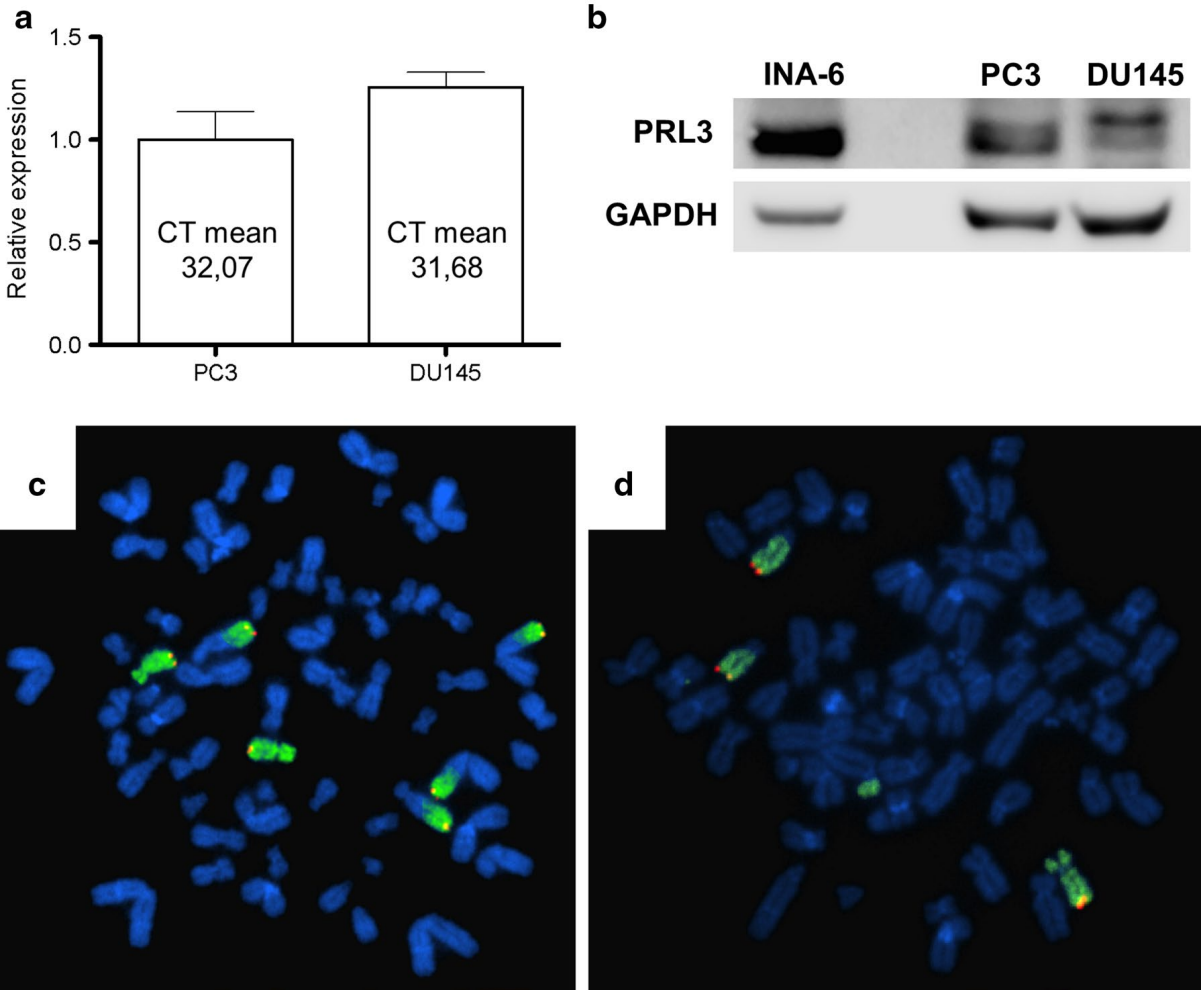
Expression of PRL-3 in prostate cancer cell lines PC3 and DU145, which have an increased copy number of *PRL*-*3* gene locus. **a** Prostate cancer cell lines PC3 and DU145 were analyzed for *PRL*-*3* expression by quantitative RT-PCR. *PRL*-*3* transcript levels were normalized to GAPDH expression in each sample and presented as relative to the *PRL*-*3* expression in the PC3 cell line, which was set to one. Mean CT values are presented in the *bars*. **b** PRL-3 protein expression was determined by Western blotting. The INA-6 cell line was used as positive control. GAPDH was used as loading control. **c**, **d** Fluorescence in situ hybridization with whole chromosome and *PRL*-*3* probe on prostate cancer cell lines PC3 (**c**) and DU145 (**d**). The *red signal marks* *PRL*-*3* (8q24.3) and the *green signal marks* the whole chromosome 8

To establish the presence of PRL-3 in our prostate cancer cell lines we used quantitative real-time PCR. The results showed that *PRL*-*3* was expressed at detectable mRNA-levels in both of the prostate cancer cell lines PC3 and DU145 (Fig. 2a). Western blots confirmed PRL-3 to be present in both cell lines (Fig. 2b). As a positive control we used the multiple myeloma cell line INA-6, which has a high endogenous levels of PRL-3 protein. In a subsequent FISH analysis, we examined whether the high expression of PRL-3 could be a result of gene amplification. PC3 cell line had two normal copies of chromosome 8 and four parts of chromosome arm 8q that had been translocated to other chromosome, resulting in six copies of *PRL*-*3* gene in total (Fig. 2c). DU145 cell line had three copies of chromosome 8 and thus three copies of the *PRL*-*3* gene (Fig. 2d).

### Inhibition of PRL-3 led to growth arrest of the prostate cancer cell lines PC3 and DU145

**Fig. 3:**
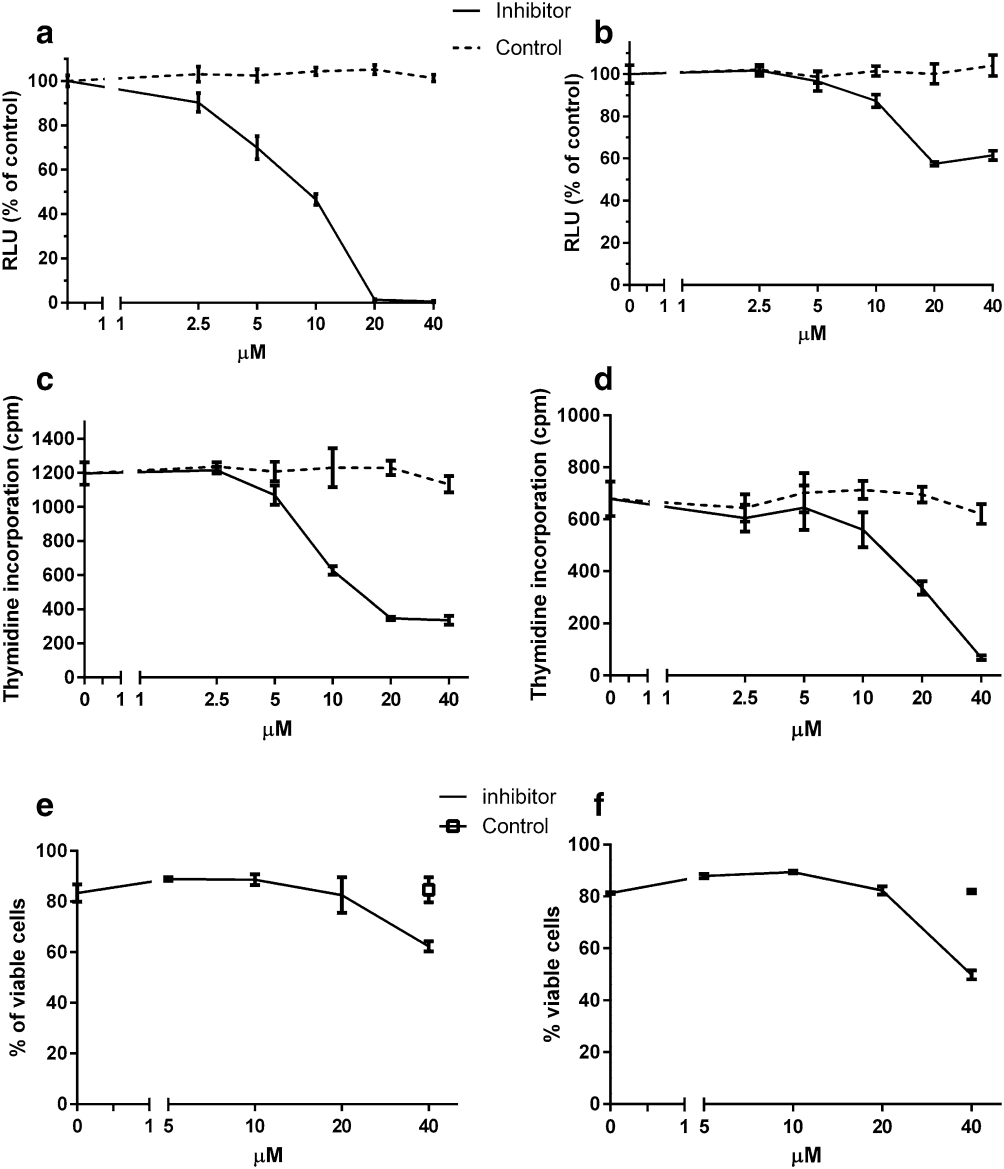
PRL-3 inhibitor I induced growth arrest and inhibited cell survival at high concentrations in prostate cancer cells. PC3 (**a** + **c**) and DU145 (**b** + **d**) cells were seeded in quintuplets in a 96-well plate, incubated in media containing 10 % FCS and with PRL-3-inhibitor I or corresponding DMSO concentrations as indicated for 72 h. Cell growth was measured with CellTiter-Glo Luminescent Cell Viability Assay (**a** + **b**) or [3H]-thymidine incorporation (**c** + **d**). Relative luciferase units (RLU) reflect the amount of ATP detected in each well. *Figure* showing one representative out of four experiments. *Error bars* represent ± one SD of quintuple measurements. Cells were incubated with media containing 10 % FCS and with various PRL-3-inhibitor I or highest DMSO concentrations for 72 h. Survival of human prostate cancer cell lines PC3 (**e**) and DU145 (**f**) was determined by annexin V-FITC/propidium iodide flow cytometry. *Figure* showing one representative of three experiments. *Error bars* represent ± one SD of duplicate measurements

We next examined whether PRL-3 was important for proliferation and protection from apoptosis in the prostate cancer cells. The effect of an inhibitor of PRL-3 was evaluated by relative ATP-measurement and 3H-thymidine incorporation. The inhibitor induced growth arrest in a dose-dependent manner in both PC3 (Fig. 3a + c) and DU145 (Fig. 3b + d) with an IC50 value of 9.9 µM (PC3, Fig. 3a) and 10.4 µM (DU145, Fig. 3b). We studied the effect of PRL-3 inhibitor on apoptosis using flow cytometry. At high concentrations (40 µM) of PRL-3 inhibitor influenced survival in the two cell lines tested (Fig. 3e, f). The PRL-3 inhibitor did not affect the survival of normal bone marrow stromal cells (BMSCs) at the concentrations used in this paper (data not shown) indicating that there was no off-target effect of the inhibitor at doses applied. Taken together, these results suggests that PRL-3 might be important for prostate cancer cell proliferation and possibly protection against apoptosis.

### Inhibition of PRL-3 decreased the migration of PC3 prostate cancer cells

**Fig. 4:**
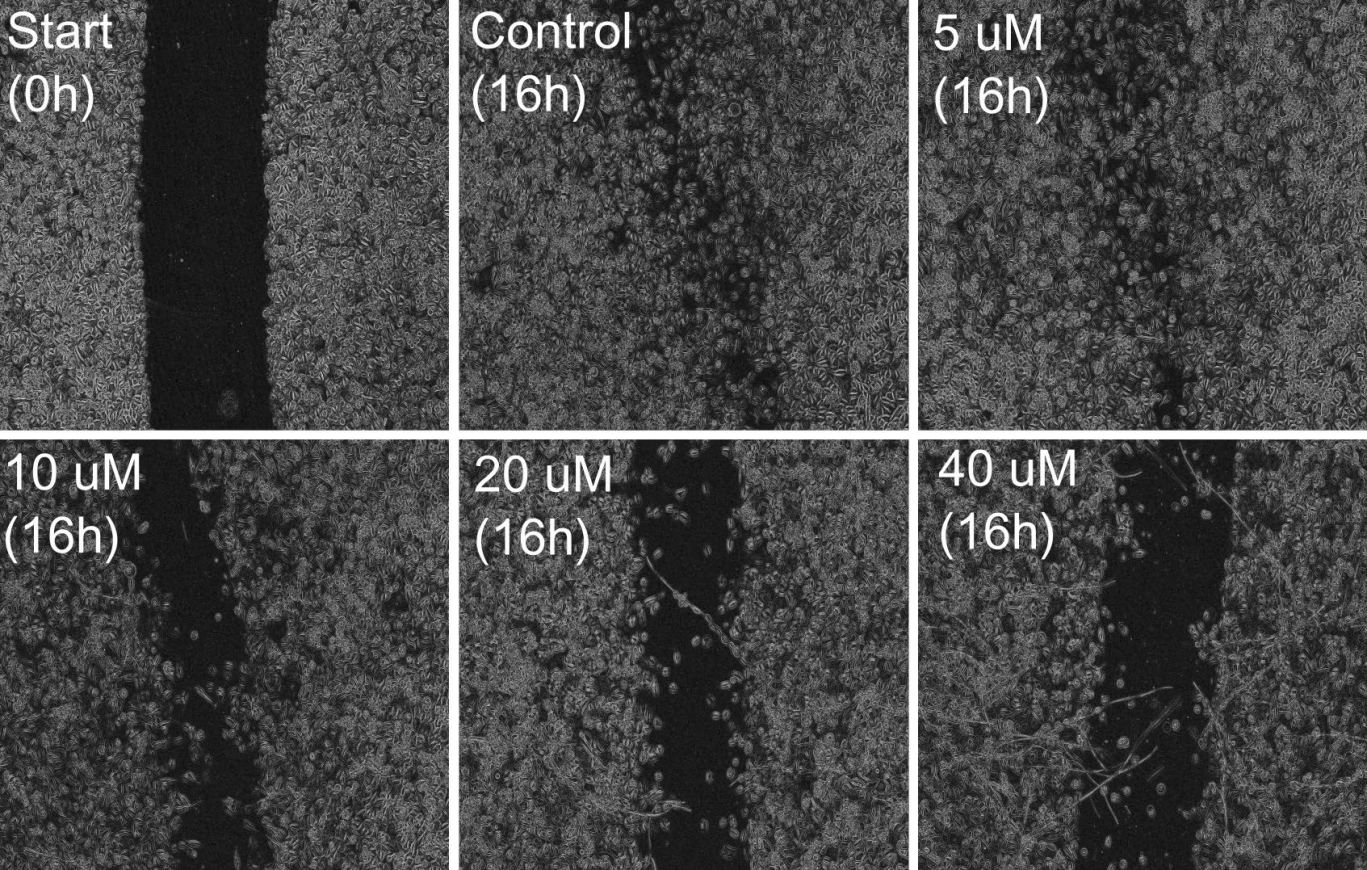
PRL-3-inhibitor I blocked the migration of the human prostate cancer cell line PC3. 10 × 10^5^ PC3 cells were seeded in each well in 6-well trays with 10 % FCS in RPMI for 48 h to grow a confluent layer. A *scratch* was made at 0 h through the confluent layer with the tip of a pipette, and then the cells were washed carefully with PBS to remove non-adherent cells. Serum free media with indicated concentrations of PRL-3 inhibitor I or DMSO (control) were added, and the cells incubated for 16 h

We further evaluated the effect of PRL-3 inhibitor I on migration in an in vitro scratch assay. As shown in Fig. 4 there was a reduction in the migratory ability of the PC3 prostate cancer cells when we inhibited PRL-3, and the inhibition of migration seemed to increase with increasing concentrations of the inhibitor. The lowest concentration of PRL-inhibitor I sufficient to provide a visual effect on the migratory ability of the cells was between 5 and 10 µM.

### PRL-3 was highly expressed in prostate cancer tumor samples and corresponding metastases

**Fig. 5:**
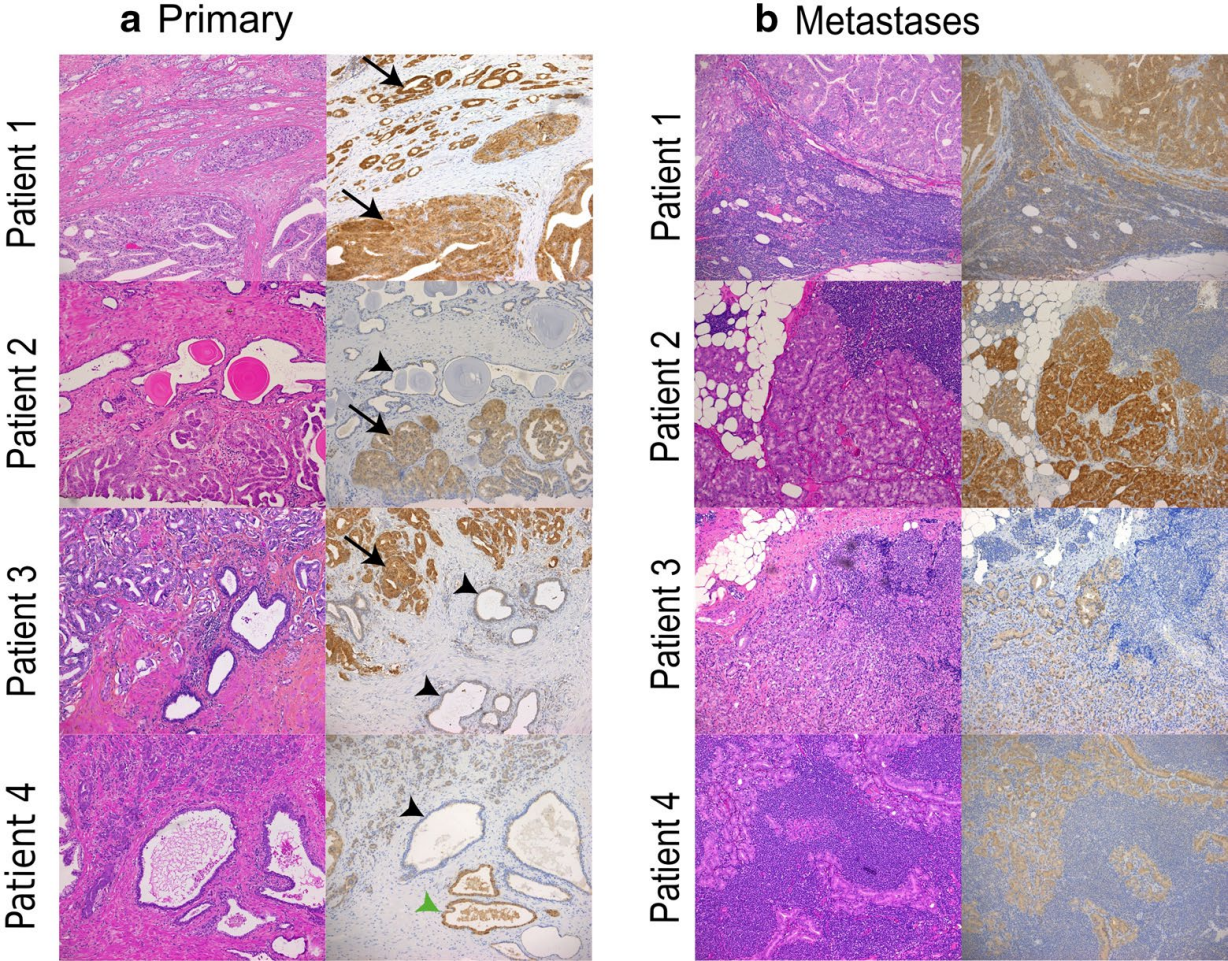
Expression of PRL-3 in primary tumors and lymph node metastasis in prostate cancer tissue samples. Immunohistochemistry staining of primary prostate cancer tumor (**a**—*Patient* 1–4) with corresponding metastases (**b**—*Patient* 1–4). 100× magnification H&E = Hematoxylin and eosin stain **a** *Patient 1* Gleason pattern 3 and 4, *Patient 2* Gleason pattern 4, *Patient 3* Gleason pattern 3 and 4, and *Patient 4* Gleason pattern 3 and 4. *Patient 2*, *3* and *4* also has histologically normal glands in the sample (*arrowheads*). IHC show PRL-3 expression in malignant glands, some histologically normal glands are also positive (*green arrowhead*), whereas others are negative (*black arrowheads*). **b** All corresponding lymph node metastases express PRL-3

To evaluate whether PRL-3 was expressed on protein level in prostate cancer, we performed immunohistochemistry on four patient samples and corresponding lymph node metastases. All primary tumors expressed PRL-3 in morphologically malignant parts of the samples (Fig. 5a), but some normal glands also stained positive (Fig. 5a, Patient 4). The corresponding metastases stained positive for PRL-3 (Fig. 5b).

## Discussion

To our knowledge, this is the first article demonstrating a pathogenetic role for PRL-3 in prostate cancer. We found a significant difference in mRNA level between normal samples and samples with malign phenotype. We investigated the functional importance of this finding with several in vitro assays. Our results indicated that PRL-3 could have a role in the proliferation and migration of prostate cancer cells, and thus could contribute to carcinogenesis and the formation of metastases. Research done in the last decade shows that *PRL*-*3* is overexpressed in carcinomas and predicts poor prognosis [8–11, 23].

We performed gene expression profiling on 156 samples from 41 patients to check for differences in expression of *PRL*-*3*. The data showed that *PRL*-*3* was differentially expressed in normal tissue compared to malignant tissue, but there was no significant difference between the low-, intermediate- or high-grade samples in these data. This is in contrast to other studies finding a positive correlation between *PRL*-*3* expression and degree of malignancy [2–8, 10–12]. Since all our samples were from patients with cancer, even the samples considered as having a normal histology could in fact contain molecular changes associated with cancer. In the samples evaluated with immunohistochemistry, PRL-3 was expressed in tissue from primary prostate cancer tumors, which is in accordance with Wang et al. [24], who performed a screening of different human cancers for protein expression of PRL-3 using immunohistochemistry, and found PRL-3 protein in 5/53 (9.4 %) prostate cancer samples. We also found the corresponding lymph node metastases to express PRL-3 protein. In our immunohistochemistry analysis there was no apparent difference of PRL-3 protein expression when comparing metastatic tissue with primary tumor tissue. This could indicate that the change in PRL-3 expression happens early (i.e. before metastatic spread) in the molecular development of prostate cancer. This does not correspond with studies on other carcinomas, where PRL-3 was found to be more specifically related to the metastatic spread from the primary tumor [7, 9, 25], although some articles proposes that PRL-3 is involved the early development of cancer [26, 27].

By inhibiting PRL-3, we induced growth arrest in the cell lines DU145 and PC3. Only at 40 µM did we see a significant effect on cell survival assessed with annexin V-FITC and PI. This in accordance with both data (unpublished) from our lab on normal bone marrow stromal cells and other studies using the same inhibitor on other cell types [28]. There are many studies investigating the effects of PRL-3 on proliferation. Most of them find that PRL-3 promotes proliferation [29–32], although results published by Basak et al. [33] indicates that both increased and reduced PRL-3 levels can inhibit proliferation. This suggests that there is a dose–response relationship between the level of PRL-3 and cellular effects, and that the effects of PRL-3 may be cell-type dependent. In our in vitro cell scratch assay, we assessed the importance of PRL-3 in migration, and found a clear reduction when inhibiting PRL-3 in the cell line PC3. This is consisted with other studies, in which the same inhibitor was used [28, 34]. The reduction in migration gives a functional explanation and supports the concept that PRL-3 may play a role in the development of metastatic disease.

Increased copy number of a gene can increase the expressed level of the particular protein that the gene encodes. According to our FISH analysis both PC3 and DU145 had increased copy number of *PRL*-*3*, although there was no clear connection between level of *PRL*-*3* expression and copy-number variations. This result is similar to a study on multiple myeloma [35], which did not find any consistent relation between number of gene copies and level of *PRL*-*3* mRNA. However, since our study is limited by the use of only two cell lines, this does not rule out that genetic copy number alterations could be important for the expression of PRL-3 in prostate cancer. Shin et al. [36] used the prostate cancer cell line PC3 to investigate the potential antimetastatic effect of a trisoxazole macrolide named halichondramide. Administration of halichondramide led to a concentration-dependent inhibition of both migration and invasion of the cells, and the suggested mechanism was suppression of PRL-3. The gene for PRL-3, *PTP4A3*, was identified as a possible factor involved in tumor progression in prostate cancer by comparing mRNA-expression and copy number alterations [18]. This article reports significantly elevated expression in cancer samples compared to benign prostate hyperplasia (BPH), but no difference between non-aggressive (Gleason score < 8) and aggressive (Gleason score ≥ 8) tumor samples. The three groups were also compared based on copy number (CN) alterations of *PRL*-*3*, and there was a significant difference between aggressive (8/17 had CN gain) and non-aggressive (1/14 had CN gain) samples. In addition, none of the 16 BPH samples analyzed had CN gain. When combining their own data with online databases (such as Oncomine), PRL-3 was found to be one of two possible markers for aggressive prostate cancer. In colorectal cancer, gene amplification accounts for some overexpression [37], but in most cases an increased transcriptional activity could explain the increased expression. Ooki et al. [38] looked at samples from primary gastric tumors, and found genomic amplification to be present in 20 % of PRL-3-positive tumor samples, and that this amplification negatively affected the outcome of the patients. Several studies have shown that PRL-3 expression is influenced by soluble factors in the tumor microenvironment [35, 39, 40]. The full mechanism of how PRL-3 is regulated is still not known. The importance and contributing factor of the various mechanisms seems to vary with cancer type.

There is little published on the function of PRL-3 in normal tissue. PRL-3 protein is detected in tissue from fetal rat hearts, but not in adult rat heart, and not in adult human heart [24]. Guo et al. [24] also found that PRL-3 protein is non-detectable in mature red blood cells and mature blood vessels. Despite the lack of literature on PRL-3 in mature tissues, heart or skeletal muscle have been described as positive controls for PRL-3 immunohistochemistry among antibody producers, indicating PRL-3 protein expression in these tissues. The possible lack of PRL-3 in normal tissue makes a PRL-3 inhibitor an attractive agent in cancer treatment. Our microarray data confirm that *PRL*-*3* is differentially expressed in cancer tissue versus normal tissue. This supports a role for PRL-3 in prostate carcinogenesis, but not specifically to metastasis. This might reflect that PRL-3 acts differently in prostate cancer than in other carcinomas, where it often is more specific for metastatic lesions. A quite small group of cancer patients and no healthy control limits our study. Nonetheless, the increased proliferation and migration due to PRL-3, and the increased expression of *PRL*-*3* mRNA in malignant samples from cancer patients, indicates that PRL-3 inhibition could be a reasonable strategy in prostate cancer treatment.

## Conclusions

This is the first study to investigate the functional role of PRL-3 in prostate cancer. We found that PRL-3 is present on both mRNA and protein level in human prostate cancer samples and corresponding lymph node metastases, and that the expression of mRNA is higher in malignant samples compared to normal samples. PRL-3 was also present on both mRNA and protein levels in the prostate cancer cell lines DU145 and PC3, and inhibition of PRL-3 by a small molecular inhibitor induced growth arrest and inhibited migration in the prostate cancer cells in a dose-dependent manner. These results suggest that PRL-3 could be a potential therapeutic target in prostate cancer.

## Additional file

10.1186/s12967-016-0830-z Further explanation of method used to adjust for stromal contribution to the samples in the gene expression profiling. P values for the four probes used in the tissue microarray analysis. All P values are compared to the samples classified as normal.

## Abbreviations

PRL-3: phosphatase of regenerating liver 3
CN: copy number
FCS: fetal calf serum
HBSS: Hanks’ balanced salt serum
FISH: fluorescence in situ hybridization
FITC: fluorescein isothiocyanate
PI: propidium iodide
