# Supplementary material for: Phosphatase of regenerating liver 3 (PRL-3) is overexpressed in human prostate cancer tissue and promotes growth and migration
Source: J Transl Med. 2016 Mar 15;14:71. doi: 10.1186/s12967-016-0830-z (PMC4791872; doi:10.1186/s12967-016-0830-z)
Supplement: Supplementary file 1 — 10.1186/s12967-016-0830-z Further explanation of method used to adjust for stromal contribution to the samples in the gene expression profiling. P values for the four probes used in the tissue microarray analysis. All P values are compared to the samples classified as normal. [file 12967_2016_830_MOESM1_ESM.pdf]

Supplementary

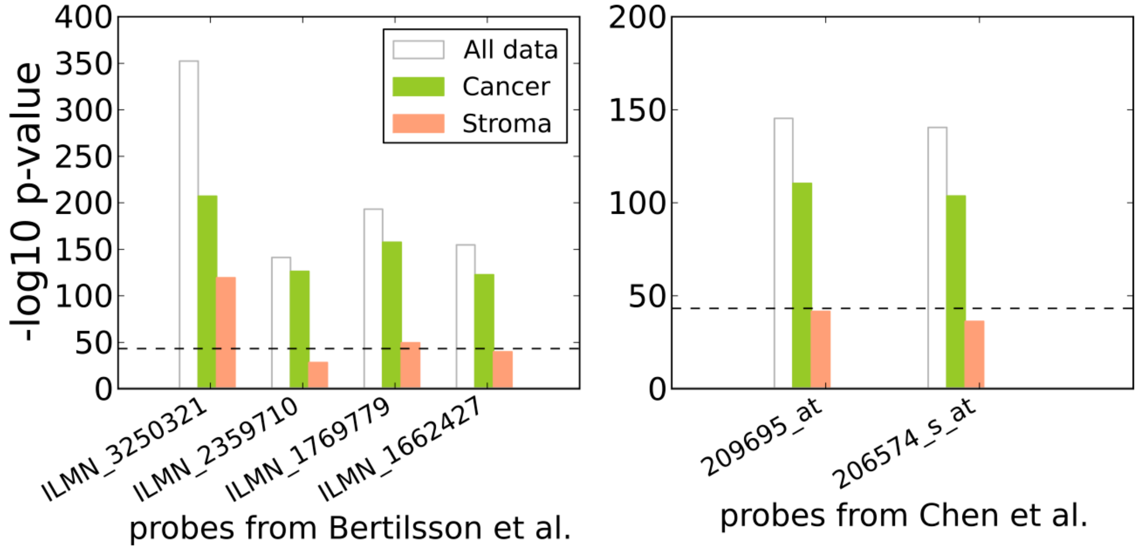

Significant expression changes for the entire dataset *(All data*; White bars) are separated into contributions from cancer related changes (*Cancer*; Green bars) and changes related to variations in stroma content (*Stroma*; Red bars). The dotted line represents a p-value of 0.05. The results are based on a strategy where the sample set is divided into two groups which, minimize and maximize the effect of the confounding stroma tissue, respectively (1). All four probes for PRL3 (*PTP4A3*) in the dataset from Bertilsson et al. (2) display an elevated cancer compared to stroma contribution. This trend was also confirmed for both *PTP4A3* probes in an independent dataset from Chen et al. (3), using the same analysis strategy. When all probes are sorted according to their respective p-values, the highest probe ranking increased from 1037 and 2832 (All data) to 64 and 122 (Cancer) in the data from Bertilsson et al. (2) and Chen et al. (3) respectively. Thus *PRL-3* rank among the top 125 cancer related genes in two independent patient cohorts, which should make it a highly relevant target in prostate cancer.

P-values for the four probes used in the tissue microarray analysis

Probe	Low vs Normal	Medium vs Normal	High vs Normal
ILMN_3250321	5.7e-8	2.0e-12	2.3e-6
ILMN_2359710	0.02	1.3e-5	4.3e-5
ILMN_1769779	0.002	5.2e-8	6.8e-5
ILMN_1662427	0.02	1.5e-6	3.1e-5

1. Rye M, Bertilsson H, Angelsen A, Bathen TF, Drabløs F, Tessem MB. A balanced tissue composition reveals new metabolic and gene expression markers in prostate cancer. Submitted.
2. Bertilsson H, Tessem MB, Flatberg A, Viset T, Gribbestad I, Angelsen A, Halgunset J. Changes in Gene Transcription Underlying the Aberrant Citrate and Choline Metabolism in Human Prostate Cancer Samples. Clin Cancer Res 2012;18(12):3261-3269.
3. Chen X, Xu S, McClelland M, Rahmatpanah F, Sawyers A, Jia Z, Mercola D. An accurate prostate cancer prognosticator using a seven-gene signature plus Gleason score and taking cell type heterogeneity into account. PLoS One 2012;7(9):e45178.
